# Insecticide susceptibility of the sand fly leishmaniasis vector *Phlebotomus argentipes* in Sri Lanka

**DOI:** 10.1186/s13071-020-04117-y

**Published:** 2020-05-13

**Authors:** Dulani R. K. Pathirage, S. H. P. Parakrama Karunaratne, Sanath C. Senanayake, Nadira D. Karunaweera

**Affiliations:** 1 Department of Parasitology, Faculty of Medicine, University of Colombo, Colombo, Sri Lanka; 2 Department of Zoology, Faculty of Science, University of Peradeniya, Peradeniya, Sri Lanka

**Keywords:** Sand fly, Insects, Insecticide resistance, *kdr* mutation, Biochemical analysis, Genetic mutation, *VGSC* gene, Bioassay, Vector control

## Abstract

**Background:**

*Leishmania donovani*-induced and sand fly-transmitted leishmaniasis is a growing health problem in Sri Lanka. Limited knowledge on biological and behavioral characteristics of probable vector *Phlebotomus argentipes* hinders disease control. Here, insecticide susceptibility patterns of *P. argentipes* were investigated with exploration of probable underlying resistance mechanisms.

**Methods:**

Adult sand flies were collected using standard cattle baited net traps and CDC light traps from selected sites in four districts. Adult F1 progeny of *P. argentipes* were exposed to different concentrations of DDT, malathion, deltamethrin and propoxur using WHO susceptibility bioassay kits. Post-1-h knockdown and post-24-h mortality were recorded and analyzed. Metabolic enzyme activity and the sensitivity of the acetylcholinesterase target-site were determined by biochemical assays using wild-caught flies. Extracted fly DNA samples were tested for the presence of knockdown-resistance (*kdr*) type mutations.

**Results:**

The LC_100_ values for DDT, malathion, propoxur and deltamethrin were 0.8–1.5%, 0.9–2.0%, 0.017–0.03% and 0.007% respectively. Insecticide**-**susceptibility levels were higher than the discriminating dosages established for *Aedes* mosquitoes, except for malathion. The lowest susceptibility levels (except for deltamethrin) were detected in the Mamadala population, whereas the highest levels were detected in the Mirigama population. The percentage of knocked-down sand flies was < 75% at any tested concentration, including those, which exhibited 100% mortality after 24 h. Elevated activity levels of glutathione S-transferase (3%, 7%, 12.5% and 14%) and esterase (2%, 5%, 5.5% and 6.5%) were detected in flies that originated from Mirigama, Pannala, Thalawa and Mamadala respectively, while monooxygenase quantities remained below the cut-off level. Ten to 34.5% of flies were heterozygous for acetylcholinesterases target-site insensitivity, associated with organophosphate and carbamate resistance. Pyrethroid-resistance-associated L1014F *kdr*-type mutation in the voltage gated sodium channel gene was detected in 30/53 flies.

**Conclusions:**

Populations of *P. argentipes* in Sri Lanka are largely susceptible to common insecticides, except for malathion (used extensively in the past for malaria control). Their insecticide susceptibility appears negatively associated with past malaria endemicity of the study sites, with signs of early insecticide tolerance. Presence of insecticide target site insensitivity in a notable proportion of flies and enhanced insecticide metabolizing enzyme activities imply potential future challenges for leishmaniasis control, with a call for urgent proactive measures for its containment.
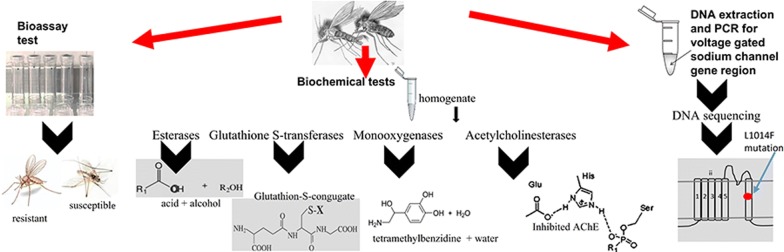

## Background

Phlebotominae sand flies are the vectors of leishmaniasis, a disease which extends to over 98 countries across the world [1]. Cutaneous leishmaniasis (CL), muco-cutaneous leishmaniasis (MCL) and visceral leishmaniasis (VL) or “kala-azar” are the three main clinical forms of the disease. The causative agent is an obligate intracellular flagellated protozoan belonging to the genus *Leishmania*. *Phlebotomus argentipes* (Diptera: Psycodidae) is the known vector of *Leishmania donovani*, the causative agent of leishmaniasis in Sri Lanka [2, 3]. The same causative species of parasite exists in India, Nepal and Bangladesh, with VL as the recognized disease [4]. Leishmaniasis is a rapidly growing health threat in Sri Lanka [5] with no national control programme yet in place to contain the situation. Among the three types of the disease, VL is the most virulent form and accounts for the second largest number of deaths due to parasitic causes [4]. The majority of *L. donovani* infections in Sri Lanka manifest as CL with only a few MCL and VL cases [6]. Although it was an exclusively imported disease prior to 1990s [7], it is now a widely prevalent disease, with case numbers increasing since 2001 and has been considered ‘notifiable’ in the health sector since 2008 [6, 8].

Control of leishmaniasis is achieved through the interruption of the cycle of transmission. Widely used methods include early infection detection and treatment of patients and control of the vector and its reservoir hosts. Vector control, although often used as a strategy for leishmaniasis control, is restricted due to the difficulties of locating the larval habitats of sand flies [9]. Therefore, vector control mostly relies on the control of adult flies often through the use of chemical insecticides. Four groups of chemical insecticides, i.e. organochlorines, organophosphates, carbamates and pyrethroids are generally used for vector control [10]. However, insecticide use is advocated only after careful studies on biological properties of the vector, including its resting behavior and insecticide susceptibility patterns. Haphazard or over-use of such chemicals often leads to development of tolerance against those agents.

Insecticides continue to be used in Sri Lanka for mosquito control, with extensive use in the past for anti-malaria activities (pre-elimination phase), which was in the form of regular indoor residual spraying programmes that continued until 2012 [11]. Therefore, sand flies may have been regularly exposed to insecticides, at least in malaria endemic areas in the dry zone, which covers two-thirds of the country [12]. Insecticide resistance in sand flies has been reported in several geographical regions around the world, particularly in malaria endemic areas, including the neighboring country India [13]. In India, DDT was replaced with pyrethroids for indoor residual spraying due to its apparent ineffectiveness in the control of VL, especially in Bihar [14, 15]. Resistance to deltamethrin has also been reported in Pondicherry, India [16]. DDT usage for indoor residual spraying programmes for the control of visceral leishmaniasis vectors in Bangladesh was banned in 1998 [17] and in Nepal 1995 [18] due to its hazardous effects. Thereafter, synthetic pyrethroids were successfully introduced, in Nepal and Bangladesh in 1992 and 2012, respectively [19]. It is believed to have contributed to the achievement of VL-elimination in Bangladesh by 2017, reaching the World Health Organization’s (WHO) set target of less than one case per 10,000 population [20] with the sand flies largely remaining susceptible to the insecticides in use [21].

Insects can resist toxic insecticidal compounds through several physiological mechanisms, including interference with enzyme systems *via* production of detoxifying enzymes and/or effects on target site(s) of the insecticide, rendering them insensitive. The neurotransmitter acetylcholine is broken down by acetylcholinesterase (AChE), which is the target site for the insecticides organophosphates and carbamates. Therefore, mutations in the AChE gene can bring about resistance to these insecticides [10, 22].

Sodium channel regulatory protein is the target site of pyrethroids and organochlorines (DDT and its analogues) and resistance to these groups of insecticides can develop due to the mutations in voltage gated sodium channel (*VGSC*) genes leading to knockdown resistance (*kdr*) [23]. If this resistance occurs at higher levels, it is known as super-*kdr* [24, 25]. To date, more than 37 resistance-associated ‛*kdr*’-type mutations or combination of mutations have been detected in pyrethroid and DDT-resistant insect populations [26, 27]. Recently two *kdr* mutations at the codon 1014 including both L1014F and L1014S have been discovered in phlebotominae sand flies in India, which are located in the same codon regions as described in mosquitoes [28].

The present study describes for the first time, to our knowledge, the insecticide susceptibility status of *P. argentipes* in Sri Lanka with investigations into the probable underlying metabolic and genetic mechanisms that may confer insecticide resistance.

## Methods

### Collection and rearing of sand flies

**Fig. 1:**
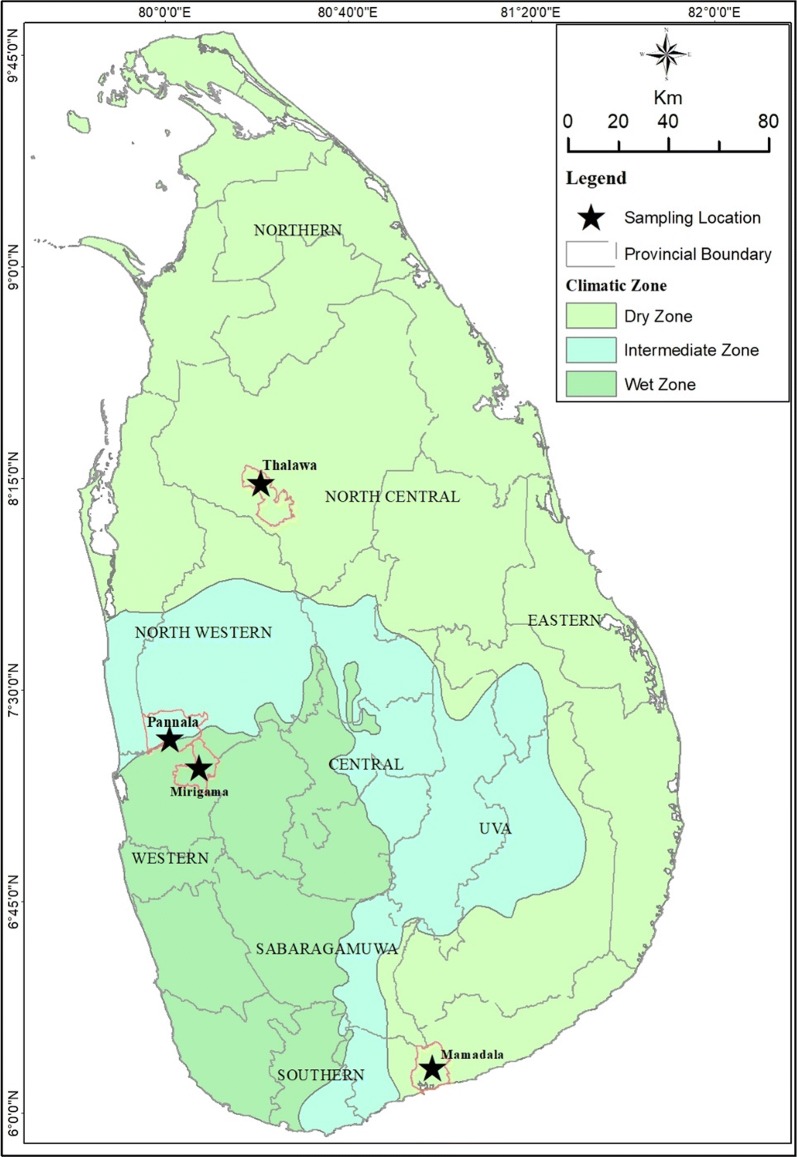
Map of Sri Lanka showing the locations of the sand fly collection sites

Sand fly adults of both sexes were collected in four administrative districts of Sri Lanka: Thalawa (8°14′11.468″N, 80°21′2.782″E) in Anuradhapura district (North-Central Province), Pannala (7°19′43.608″N, 80°1′26.3316″E) in Kurunegala district (North-Western Province), Mamadala (6°07′16.80″N, 81°07′12.60″E) in Hambantota district (Southern Province), and Mirigama (7°13′30.72″N, 80°7′40.439″E) in Gampaha district (Western Province) (Fig. 1). All collections were made between November 2015 and November 2017. The country is arbitrarily divided into dry, intermediate and wet zones based on the annual rainfall. Thalawa and Mamadala are located within the dry zone, Pannala in the intermediate zone and Mirigama in the wet zone (Fig. 1). Study sites were selected based on leishmaniasis case burden [5], vector prevalence [29] and to represent different climatic zones [30]. At each site, the standard cattle-baited net traps [31] and Center for Disease Control (CDC) light traps were used for sand fly collections. Traps were set at 18:00 h each evening and flies were collected each morning at 6:00 h.

Sand flies were transferred by aspirators from trap nets into plaster of Paris-lined plastic pots covered with a piece of net. A hole in the net (for insertion of the aspirator) was covered with a cotton swab soaked with 30% sucrose solution to feed sand flies. The samples were then transferred to the insectary of the Department of Parasitology, Faculty of Medicine, University of Colombo, Sri Lanka, for rearing. The methods of Volf & Volfova [32] were used thereafter, for isolation of newly emerged flies (F1 progeny) for insecticide bioassay tests. Identification was carried out to the species level by examination under a light microscope at a magnification of 400× using standard taxonomic keys [33, 34].

### Insecticides, chemicals and equipment

Chemicals were purchased from Sigma-Aldrich (Poole, UK) unless otherwise stated. DDT (an organochlorine), malathion (an organophosphate), deltamethrin (a pyrethroid) and propoxur (a carbamate) (97–99% pure) were obtained from ChemService (Maidstone, UK). Biochemical assay plates were read using an EPOCH 2 absorbance microtitre plate reader with Gen 5.03 software (Molecular Devices, Bio-Tek, Vermont, USA) Protein assay kit (Bio-Rad, Hemel Hempstead, UK) was used for the detection of protein content in fly homogenates.

### Insecticide-impregnated papers and insecticide bioassays

Insecticide-impregnated papers were prepared at the Department of Zoology, Faculty of Science, University of Peradeniya, Sri Lanka, as previously described [35], using the standard WHO method [36]. In brief, the technical grade insecticides at a pre-determined range of concentrations were mixed with acetone (as the spreading agent). DDT (0.4–4.0%), malathion (0.5–5.0%) and propoxur (0.01–0.10%) solutions were dissolved in olive oil. Deltamethrin (0.005–0.050%) solutions were prepared in Dow-Corning 556 silicone fluid. Rectangles of Whatman No. 1 filter paper (12 × 15 cm) were used for insecticide impregnation. Insecticide/oil solutions (0.7 ml) were mixed with an equal volume of acetone and the mixture was applied uniformly on the filter paper. Acetone enabled an even distribution of the solution on the paper. Insecticide-impregnated papers were left overnight at room temperature to allow complete evaporation of acetone. Papers were then foil wrapped and stored at − 20 °C until use. Each paper was used for repeat experiments, but only up to 5 times [36].

Insecticide bioassays were performed using standard WHO procedures [37] to investigate the susceptibility pattern of *P. argentipes* for DDT, malathion, deltamethrin and propoxur insecticides. In each test, 20 female sand flies from the F1 population were placed in a holding tube lined with an insecticide-free paper for 0.5 h at 26 ± 1 °C to acclimatize. Then they were transferred to the testing tube with insecticide-impregnated filter paper. The experimental tubes were allowed to stand upright for 1 h; insects were then transferred to holding tubes for a further 24 h recovery period with 30% sugar at 26 ± 1 °C. Five replicates were used for each concentration of insecticide. The percentage of flies knocked-down after one hour of insecticide exposure and the percentage mortality after 24 h recovery period were calculated. Papers with oil and acetone were used as the negative controls for each experiment. Data were considered only when the mortality in the control tubes were less than 20%, and the results were validated with the control samples using Abbott’s formula: [(% test mortality − % control mortality) × 100]/[100 − % control mortality)] [38].

### Biochemical assays

WHO-prescribed biochemical tests were performed, with slight modifications [39]. Specific activity levels of esterase, glutathione S-transferase and the amounts of monooxygenases were determined in two-hundred wild-caught adult sand flies from each study site [39]. Each fly was separately homogenized in 100 µl of ice-cold distilled water. Two replicates of 10 µl of a crude homogenate obtained after centrifugation at 13,000× *rpm* for 2 min were used for each of the above standard assays.

Another 200 flies from each study site were homogenized individually in 70 µl of ice-cold distilled water to assess the sensitivity of the acetylcholinesterase (AChE). Two replicates of 25 μl of crude homogenate were used [39].

### Detection of *kdr* mutations

DNA was extracted using DNeasy® Blood and Tissue kit (Qiagen, Hilden, Germany) from individual flies with a total of 10–15 *P. argentipes* flies per site. Homogenates were incubated overnight after adding proteinase K at 56 °C until tissues were completely lysed.

PCR amplification of domain II of the *VGSC* gene was carried out using primers Vssc8F (5′-AAT GTG GGA TTG CAT GCT GG-3′) and Vssc1bR (5′-CGT ATC ATT GTC TGC AGT TGG T-3′) [28] in a 50 µl PCR reaction which contained 2 µl of each primer, 10 µl of 5× Colourless GoTaq® Flexi Reaction Buffer (Promega, Wisconsin, USA), 4 µl of 25 mM MgCl_2,_ 1 µl of 10 mM of each dNTP and 5 units of GoTaq® Flexi DNA polymerase (Promega). PCR cycling conditions included an initial denaturation step of 5 min at 95 °C followed by 30 cycles each of 96 °C for 30 s, 56 °C for 30 s, and 72 °C for 30 s, and a final extension step at 72 °C for 5 min. PCR products were electrophoresed on a 1% agarose gel stained with ethidium bromide. Amplified PCR products were sent to Macrogen, Korea for purification and DNA sequencing using an Applied Biosystems 3730 DNA Analyzer. The sequences (GenBank: MN685050-MN685102) were analyzed using BioEdit software v.7 (http://www.mbio.ncsu.edu/BioEdit/bioedit.html) to determine the possible mutations of the *VGSC* gene that confer resistance at the pyrethroid target site sodium channel regulatory proteins (*kdr-*type resistance). Amplified domain II *VGSC* gene sequences were further analyzed and compared with the NCBI database *VGSC* domain II sequence of *Musca domestica* (GenBank: AAB47604).

### Data analysis

The concentration *versus* mortality and knockdown relationships were determined for DDT, malathion, deltamethrin and propoxur using bar charts in Sigma Plot (version 10). Wilcoxonʼs signed-rank test was used to test whether the results are significantly different among the four populations for each insecticide. Boxplots were prepared using Minitab (version 15) for each insecticide to test significant differences using ANOVA of elevated enzymes and to determine the remaining enzyme activities of flies in each location. Allelic frequencies with L1014F mutation of flies in each location were calculated.

## Results

### Insecticide bioassays

**Fig. 2:**
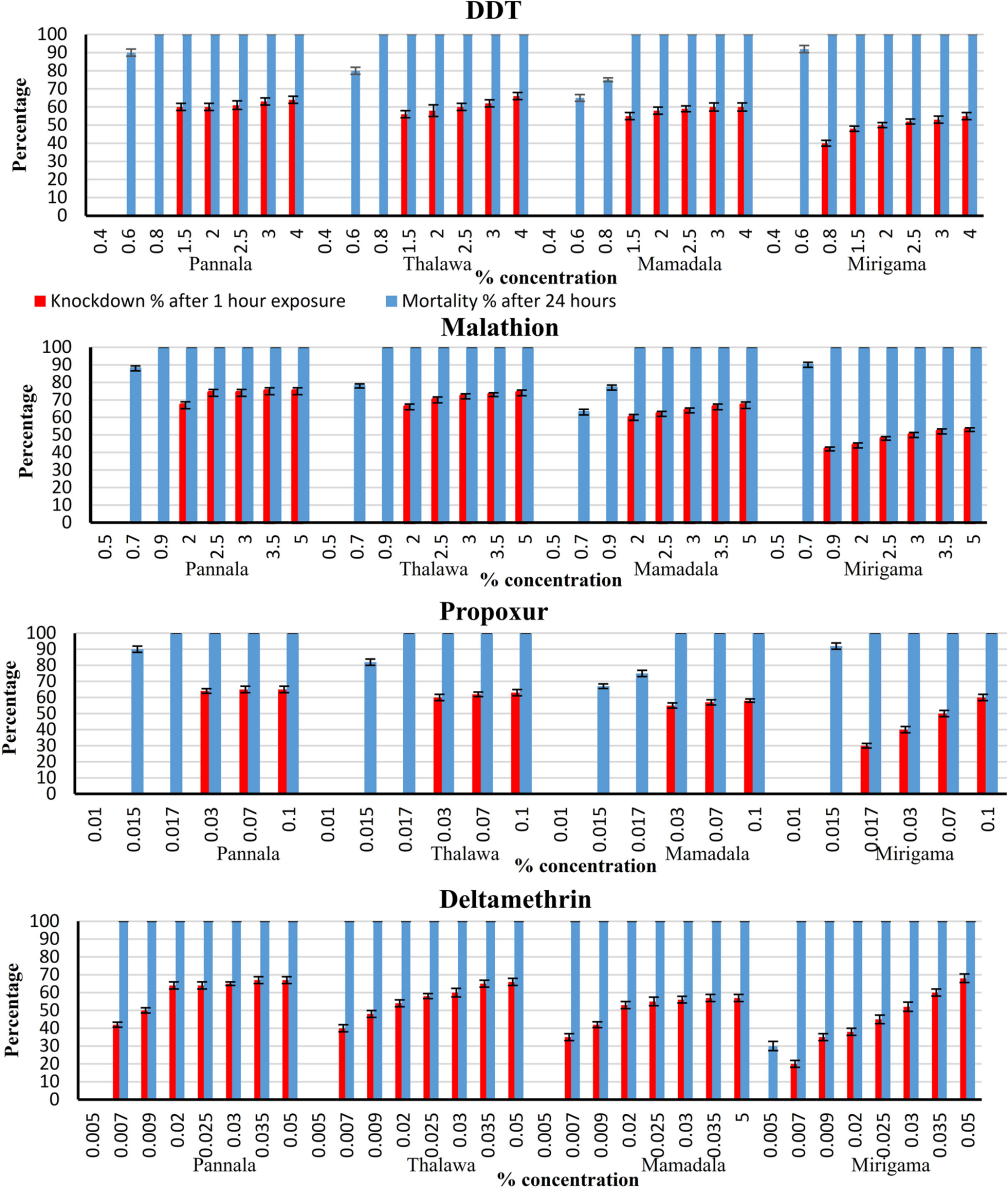
Knockdown and mortality percentages in susceptibility tests using the WHO bioassays with *Phlebotomus argentipes* populations from Pannala, Thalawa, Mamadala and Mirigama for different concentrations of DDT, malathion, propoxur and deltamethrin. *n* = 100 flies for each concentration of insecticide. The knockdown percentages are given at 1 h after exposure and the mortality percentages are given at 24 h after exposure. Error bars represent lower and upper 95% confidence limits

The concentration *versus* mortality and knockdown relationships obtained from the bioassay experiments are presented in Fig. 2. Although the sand flies were tested over a wide range of insecticide concentrations, the mortality percentages moved from 0% to 100% within a very narrow range (Fig. 2; Table 1).

**Table 1:** Insecticide concentrations and associated lethal dosages for *Phlebotomus argentipes* from four study sites

Insecticide	Tested concentration^a^	Population	LC_0_^b^	LC_100_^c^
DDT	0.4, 0.6, 0.8, 1.5, 2.0, 2.5, 3.0, 4.0	Pannala	0.4	0.8
		Thalawa	0.4	0.8
		Mamadala	0.4	1.5
		Mirigama	0.4	0.8
Malathion	0.5, 0.7, 0.9, 2.0, 2.5, 3.0, 3.5, 5.0	Pannala	0.5	0.9
		Thalawa	0.5	0.9
		Mamadala	0.5	2.0
		Mirigama	0.5	0.9
Propoxur	0.01, 0.015, 0.017, 0.03, 0.07, 0.1	Pannala	0.01	0.017
		Thalawa	0.01	0.017
		Mamadala	0.01	0.03
		Mirigama	0.01	0.017
Deltamethrin	0.005, 0.007, 0.009, 0.02, 0.025, 0.03, 0.035, 0.05	Pannala	0.005	0.007
		Thalawa	0.005	0.007
		Mamadala	0.005	0.007
		Mirigama	< 0.005	0.007

^a^*n* ≥ 100 for each concentration of each insecticide

^b^Highest concentration which resulted in a mortality of 0%

^c^Lowest concentration which resulted in a mortality of 100%

Sand flies from all four field sites survived (LC_0_) the exposure to concentrations of 0.4% DDT, 0.5% malathion, 0.01% propoxur and 0.005% deltamethrin (Table 1). However, there was 100% mortality (LC_100_) of sand fly populations from all sites at 0.8% DDT, 0.9% malathion and 0.017% propoxur, except for flies from Mamadala. The Mamadala population reached 100% mortality at higher concentrations of insecticides, i.e. 1.5% DDT, 2.0% malathion and 0.03% propoxur. The lowest concentration which resulted in a mortality of 100% for deltamethrin was 0.007% across all the study sites.

DDT 0.8% and malathion 0.9% killed all flies from Pannala, Thalawa and Mirigama whereas, for the same concentrations of the insecticides, only 75% and 77% mortality, respectively, were observed for Mamadala flies. Similarly, propoxur 0.017% resulted in a mortality of 100% in Pannala, Thalawa and Mirigama populations while Mamadala had a mortality of only 75% for the same propoxur concentration. No mortality was observed for deltamethrin 0.005% in the fly populations except for the Mirigama population, where a 30% mortality was observed for 0.005% deltamethrin. However, 0.007% deltamethrin resulted in a mortality of 100% in all four populations.

Results of the statistical analysis showed that there was no significant difference in mortality between flies originating from the four populations for each of the insecticides (DDT, malathion, propoxur and deltamethrin) with no significant difference in mortality rates between specific concentrations of tested insecticides (*P > *0.05; Fig. 2). Details of statistical results are provided in Additional file 1: Table S1.

Flies that were exposed to the tested insecticides for 1 h exhibited zero knockdown at concentrations below 0.8% (DDT), 0.9% (malathion), 0.017% (propoxur) and 0.005% (deltamethrin). The maximum knockdown rate did not exceed 75%, even at concentrations which resulted in a mortality of 100% after the 24 h recovery period (Fig. 2).

### Biochemical assays

**Fig. 3:**
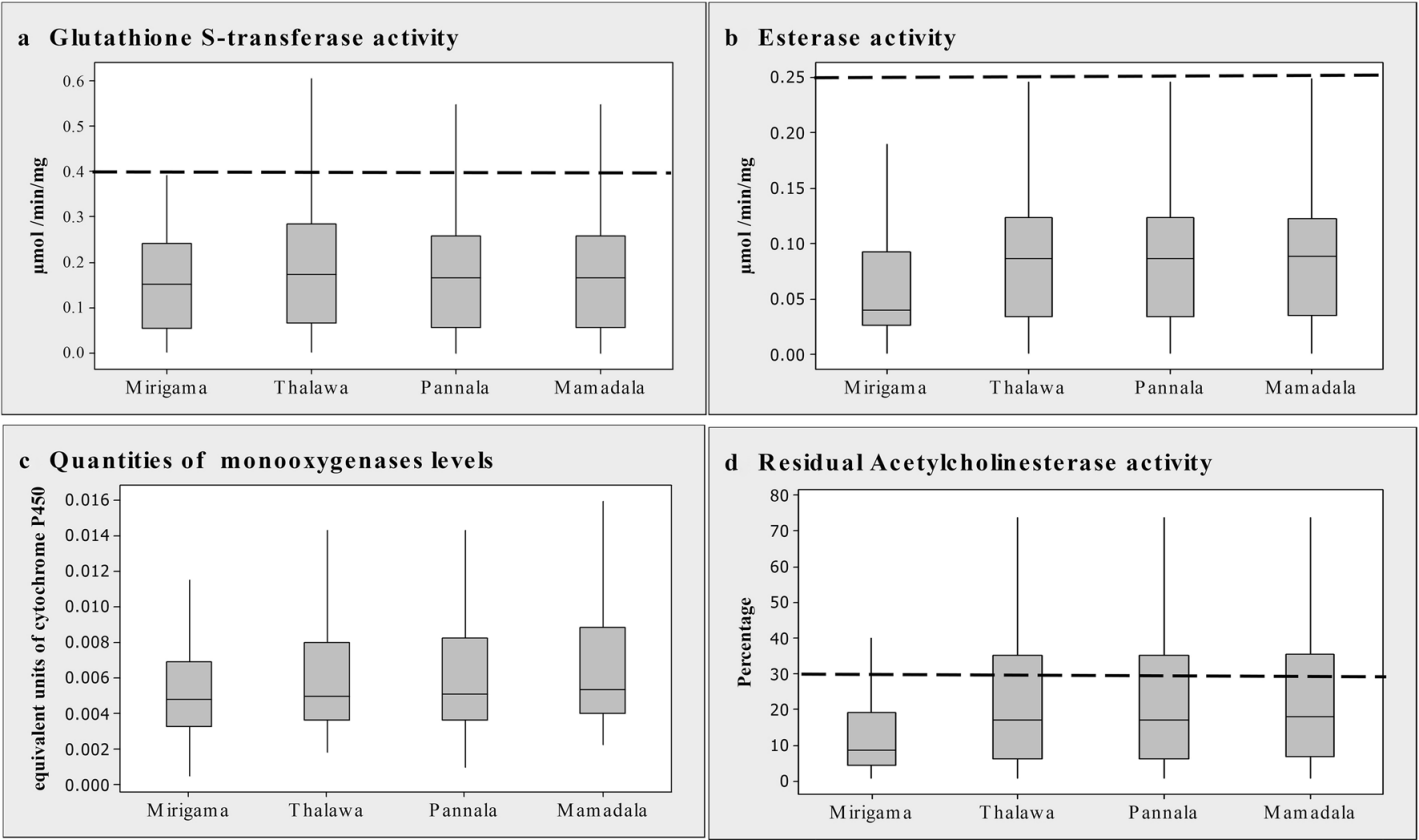
Activity levels of insecticide metabolizing enzymes. **a** Glutathione S-transferase. **b** Esterase. **c** Quantities of monooxygenases. **d** Residual acetylcholinesterase activity in *P. argentipes* originated from the study sites Mirigama, Thalawa, Pannala and Mamadala. The discriminating values for each measure as used for anopheline mosquitoes [39, 64] are shown as interrupted lines (**a**–**d**). In the case of cytochrome P450, the discriminating value of anopheline mosquitoes is at 0.35 equivalent units, therefore, is off the scale and not shown (**c**). Shaded boxes indicate the percentage of flies in each activity range of assays with the short horizontal line in each shaded box representing the mean value. The vertical lines that extend from each shaded box represent the percentage of flies that deviated from the mean activity levels at each study site

Enzyme activity profiles of glutathione S-transferase (GST) and carboxylesterase, and the quantity profiles of monooxygenases of sand flies from all four study sites are shown in Fig. 3a, b and c, respectively. Already established discriminating values for enhanced activities/amounts of these enzymes that contribute to resistance in anopheline mosquitoes are given as interrupted lines (except for cytochrome P450 monooxygenase, since its discriminating value as given for anophelines is 0.35 equivalent units, which is off the scale). Mean values of carboxylesterase and GST activities and the quantity of monooxygenase of the four sand fly populations are given in Table 2. According to the profiles and mean values, the majority of flies in each population did not show elevated activities/amounts of these detoxifying enzymes. Only 3%, 7%, 12.5% and 14% of Mirigama, Pannala, Thalawa and Mamadala populations, respectively, had GST activities higher than discriminating dosages as given for mosquitoes (0.4 µmol min^−1^ mg^−1^). Similarly, only 2%, 5%, 5.5% and 6.5%, respectively, had esterase activities higher than that value (0.25 µmol min^−1^ mg^−1^), while none of the flies had monooxygenase quantities higher than the mosquito cut-off value of 0.35 equivalent units of cytochrome P450. However, there was no significant difference in mean values of GST activity and the quantity of monooxygenases among four populations in this study (*F*_(3, 788)_ = 2.02, *P* = 0.110 and *F*_(3, 792)_ = 2.46, *P* = 0.062, respectively). Although the mean values of esterase activities were not significantly different in Mamadala, Thalawa and Pannala flies (*F*_(2, 594)_ = 0.03, *P* = 0.972), the mean esterase activity of the Mirigama population was significantly higher than that of the other three populations (*F*_(3, 792)_ = 5.00, *P* = 0.002) (Additional file 2: Text S1). Remaining AChE activity of the propoxur-inhibited crude homogenate fraction was calculated for each fly as a percentage of the activity of the uninhibited fraction. Remaining activity profiles and the average activities for all four populations are shown in Fig. 3d and Table 2. According to the WHO standards, remaining activity of < 30% indicates that the population is homozygous susceptible for insecticide inhibition whereas values of 30–70% indicate heterogeneity, and values *> *70% indicate homozygous resistance status [39]. After inhibition, less than 30% remaining activity of the AChE target site was shown by 90%, 72%, 68.5% and 65.5% of Mirigama, Pannala, Thalawa and Mamadala sand fly populations, respectively (Fig. 3d). The remaining activities of the AChE target site were comparable in the flies originating from Mamadala, Thalawa and Pannala (*F*_(2, 594)_ = 0.12, *P* = 0.890) and significantly higher when compared with flies originating from Mirigama (*F*_(3, 792)_ = 14.75, *P* < 0.0001) (Table 2, Additional file 2: Text S1).

**Table 2:** Mean activity/quantity of insecticide-detoxifying enzymes and remaining activity of propoxur-inhibited AChE in *Phlebotomus argentipes* (*n* ≥ 200)

Population	Insecticide metabolizing enzyme activity			Target site activity
	Esterase activity (µmol min^−1^ mg^−1^)	Glutathione S-transferase activity (µmol min^−1^ mg^−1^)	Monooxygenase amount^a^	AChE^b^
	Mean ± SE	Mean ± SE	Mean ± SE	Mean ± SE
Mamadala	0.108 ± 0.0093	0.198 ± 0.0145	0.0074 ± 0.0004	24.6 ± 1.690^c^
Thalawa	0.105 ± 0.0093	0.221 ± 0.0166	0.0067 ± 0.0004	24.1 ± 1.700^c^
Pannala	0.106 ± 0.0093	0.198 ± 0.0145	0.0069 ± 0.0004	23.5 ± 1.540^c^
Mirigama	0.069 ± 0.0046	0.172 ± 0.0105	0.0059 ± 0.0003	12.6 ± 0.837**

^a^Equivalent units of cytochrome P450

^b^Remaining activity of propoxur-inhibited AChE given as a percentage of the activity of un-inhibited fraction

^c^Values comparable with each other (*F*_(2, 594)_ = 0.12, *P* = 0.890)

^**^Significantly different from values in the other three sites (*F*_(3, 792)_v14.75, *P* < 0.0001)

*Abbreviation*: SE, standard error of the mean

### Detection of *kdr* mutations

The L1014 mutation, a known mutation in insects that produces resistance to pyrethroids [26, 27], was discovered in 30 out of 53 sand flies tested. It is a non-synonymous mutation substituting leucine with phenylalanine, or rarely serine at position 1014. Out of 30 mutations detected in the present study, 29 had the L1014F mutation while only one sample had the L1014S mutation (Table 3, Additional file 3: Figure S1). The highest frequency of flies having the L1014F mutation was found in Mamadala (Table 3, Additional file 3: Figure S1).

**Table 3:** Frequencies of the allele with L1014F mutation in *Phlebotomus argentipes* populations from the four study sites

Population	No. of flies tested^a^	Allelic frequency, *n* (%)^b^				
		LL	LF	FF	LS	SS
Mamadala	15	2 (13.3)	1 (6.7)	11 (73.3)	0 (0)	1 (6.7)
Thalawa	15	5 (33.3)	0 (0)	10 (66.7)	0 (0)	0 (0)
Pannala	11	5 (45.5)	2 (18.2)	4 (36.7)	0 (0)	0 (0)
Mirigama	12	10 (83.3)	0 (0)	2 (16.7)	0 (0)	0 (0)
Total	53	22 (41.5)	3 (5.7)	27 (50.9)	0 (0)	1 (1.9)

^a^Tested for L1014F mutation

^b^Allelic frequencies for L1014 mutations

*Abbreviations*: L, leucine; F, phenylalanine; S, serine; n, number of alleles

## Discussion

The study describes, to our knowledge for the first time, early signs of insecticide tolerance as evident through bioassays and biochemical investigations, and the presence of *kdr* genetic mutation in phlebotomine sand flies in Sri Lanka.

Vector control is an established strategy used to arrest leishmaniasis transmission in endemic areas, especially through regular indoor residual spraying of chemical insecticides. Such strategies are being used in the neighboring countries India, Nepal and Bangladesh, with the aim of eliminating leishmaniasis by the year 2020 [40]. The susceptibility of Sri Lankan sand flies to insecticides was studied using a standard methodology. The results revealed that the Sri Lankan sand fly populations were largely susceptible to the insecticides tested (except perhaps to malathion), when compared against the resistant discriminating dosages for mosquitoes. However, the range of concentrations of insecticides that gave values between LC_0_ and LC_100_ in sand flies was very narrow, and therefore, only a limited number of intermediate concentrations could be tested. Furthermore, regression lines for log probit mortality curves could not be obtained due to insufficient data points between 0 and 100%, which precluded the calculation of LC_50_ values.

Previous studies have also used discriminating concentrations or fixed dosages of insecticides to evaluate the susceptibility status of sand fly populations using mosquitoes as the standard [41–43]. For anopheline mosquitoes, the discriminating dosages have been recorded as 4%, 5%, 0.1% and 0.05% for DDT, malathion, propoxur and deltamethrin, respectively [44]. For aedine mosquitoes, the values are 4% DDT, 0.8% malathion, 0.1% propoxur [45] and 0.03% deltamethrin [46]. For the Sri Lankan sand fly populations tested in the present study, the lowest concentrations of insecticides that resulted in a mortality of 100% were 0.8–1.5 %, 0.9–2.0%, 0.017–0.03% and 0.007%, respectively, for the above insecticides. Therefore, the Sri Lankan populations of *P. argentipes* can be considered as susceptible to the tested insecticides, when compared against the discriminating dosages specified for either anopheline or aedine mosquitoes. The only exception was malathion for which the LC_100_ values were 1.5% for Mamadala, which was higher than the discriminating dosages given for aedine mosquitoes, i.e. 0.8% (while it was 0.8% in the other three sand fly populations tested). Similarly, sand fly populations from neighboring countries such as in Bihar State, India, and the border villages of Nepal have shown high malathion-resistance (and DDT-resistance) with ability to survive even at 5% malathion (and 4% DDT) [41]. Moreover, *Phlebotomus papatasi* from Surogia village in Khartoum State, Sudan, and *P. argentipes* from Baizalpur, Chandi and Khusroopur villages in India, have also shown such high insecticide resistance [43].

Except for deltamethrin, the LC_100_ values were relatively higher in the Mamadala sand fly population when compared to the other three populations studied. Mamadala is located within the dry zone, in the district of Hambantota (Southern Province), where high malaria transmission prevailed in the past [47] with intensive insecticide usage for mosquito control [48, 49]. Based on the country’s annual malaria incidence reports [50–54], the highest number of malaria cases have been reported from the Hambantota district, consistently over a 5-year period prior to 2012 (when malaria was eliminated) [11]. DDT was used in Sri Lanka for both agricultural insect pest control and malaria vector control, until 1975/1977 when its usage was banned due to environmental concerns and the appearance of widespread DDT resistance among malaria vectors [49, 55]. Organophosphates, mainly malathion, replaced DDT in mosquito control programmes and carbamates were introduced to the agricultural sector [49, 55]. Pyrethroids were first introduced to both health and agricultural sectors in mid-1990s [49, 55]. Such history of prolonged exposure to insecticides may be responsible for the observations made in the present study, especially the malathion resistance observed in the tested sand fly populations. It is noteworthy, that the calculations and cut-offs used in this study were based on the current recommendations and insecticide discriminating dosages described by the WHO for mosquitoes. This is due to the non-availability of either specific recommendations or resistance discriminating values for sand flies. It is tempting to speculate that the actual cut-offs for resistance discrimination in sand flies may be even lower than those given for mosquitoes, considering the smaller body mass and the softness of the cuticle of these tiny insects. However, the toxicity profile of an organism is considered species-specific and is known to be determined by a complex interplay of factors and therefore, no solid data exist to support such speculations.

It was noted that the knockdown effect, recorded as percent knockdown flies after one hour of insecticide exposure, increased with increasing concentrations of an insecticide. However, this was only up to a certain point of concentration of the insecticide, and never exceeded 75% at any of the tested concentrations, which included those that resulted in a mortality of 100% after a 24-hour recovery period. For Pannala and Thalawa populations, certain tested concentrations of DDT, malathion and propoxur showed zero knockdown after one hour of exposure, but a mortality of 100% after 24 hours. This apparent discrepancy between the knockdown concentrations and the LC values may be an early indication of the development of insecticide tolerance in these populations, as described previously [56].

According to the WHO guidelines, remaining activity of < 30% of propoxur-inhibited AChE target site is considered as the cut-off level for its sensitivity [39]. Our study indicates 10–34.5% of the tested sand fly populations were heterozygous insensitive at the AChE target site that is associated with organophosphate and carbamate resistance. A previous study in Delft Island in Sri Lanka (a small island located close to the northern border of the country) has also shown a similar high prevalence of reduced sensitivity of the AChE target site in sand flies [12]. Using the same biochemical assays Surendran et al. [12] have shown that 14% of the Delft island population of sand flies had *> *80% residual activity of AChEs after propoxur inhibition.

Results of the biochemical assays indicated that GST and esterase activities of the populations were poorly elevated. All four populations showed no elevation of monooxygenases. Sand flies from Delft island of Sri Lanka have shown a slight elevation of esterase and GST but no elevation of monooxygenases [12]. However, the impact of elevated esterases and insensitive AChE on the resistance status of sand flies was not assessed in the study of Surendran et al. [12]. Based on the present observations, it might be assumed that the development of tolerance to all four groups of insecticides is ‘in progress’ in Sri Lankan sand fly populations. This might be attributed to the prolonged and widespread use of insecticides in both agricultural and health sector insect pest/vector control programmes [49, 55, 57]. If the study sites are lined up based on the malaria case burden in the relevant district during the 2007–2012 period, the order would be as: Mirigama < Pannala < Thalawa < Mamadala [11, 50–54]. This order is reversed apparently when the trend of insecticide susceptibility of sand flies in each study site (as demonstrated in this study) is considered. However, statistically there was no significant difference in mortality among the populations studied here for the tested insecticides.

Association of the L1014F *kdr*-type mutation in pyrethroid resistance was initially discovered in house flies and cockroaches [24, 25, 58–60]. Later, the same mutation was recorded from several other pyrethroid-resistant insects [26]. In Sri Lanka, the presence of the L1014F *kdr*-type mutation has been previously reported the the mosquitoes *Culex quinquefasciatus* [61] and *Anopheles subpictus* [62] and the bed bug *Cimex hemipterus* [63]. However, no previous studies have described the presence of *kdr* type mutations in sand flies in Sri Lanka, although L1014F and L1014S *kdr*-type mutations have been recorded in the sand fly population in India [28]. It is interesting to note that the L1014F/L1014S mutation was present in a notable proportion of sand flies tested in the present study, with the highest mutation frequency evident in the Mamadala samples, presumably associated with the history of high pyrethroid spraying (N. Subasinghe, Regional Malaria Officer, Hambantota, Sri Lanka, personal communication).

Sri Lankan sand fly populations from the wild were in most part susceptible to the insecticides tested (except perhaps for malathion), when judged against the resistant discriminating dosages of mosquitoes. However, the less than optimal knockdown rates, the presence of marginally higher concentrations of insecticide detoxifying enzymes with the presence of the AChE target site insensitivity, relatively lower levels of insecticide susceptibility (except for deltamethrin) in flies originated from a location with a history of heavy insecticide usage and the presence of a known genetic mutation associated with insecticide resistance in a notable proportion of flies, could be viewed as a warning signal for the development of tolerance against insecticides.

## Conclusions

Populations of *P. argentipes* in Sri Lanka seem to be susceptible to a broad range of insecticides. However, the early signs of insecticide tolerance that were evident in a country where strategic initiatives towards vector control of leishmaniasis are not yet in place, imply substantial challenges for future leishmaniasis control. Therefore, these findings, demand a regionally-coordinated strategic plan to address the apparent threat of insecticide tolerance in local sand fly populations. Such initiatives may include regulations imposed on the use of insecticides, particularly in the agricultural sector. The findings will also pave the way for more extensive investigations to aid a vector control strategy that may need to be adopted within a future national leishmaniasis control programme.

## Supplementary information

**Additional file 1: Table S1.** Statistical analysis to test for significant differences of sand fly mortality between the studied populations to tested insecticides using the Wilcoxon signed-rank test.

**Additional file 2: Text S1.** Statistical analysis to test for significant differences using ANOVA of elevated enzymes and remaining enzyme activities in each location.

**Additional file 3: Figure S1.** Sequence alignment of the *VGSC* gene in *P. argentipes* with the sequence for the voltage-sensitive sodium channel of *Musca domestica* (GenBank: AAB47604). Arrow indicates the position of the L1014F mutation. Heterozygotes samples were indicated with an asterisk (*).

## Data Availability

Data supporting the conclusions of this article are included within the article and its additional files. The datasets used and/or analyzed during the present study are available from the corresponding author upon reasonable request.

## Abbreviations

AChE: acetylcholinesterase
CDC: Center for Disease Control
CL: cutaneous leishmaniasis
GST: glutathione S-transferase
*kdr*: knockdown resistance
LC_100_: the lowest dose, which kills 100% of the tested population after 24 hours
LC_0_: the dosage, which is not lethal to any flies in the population
MCL: muco-cutaneous leishmaniasis
VL: visceral leishmaniasis
*VGSC*: voltage gated sodium channel
WHO: World Health Organization
