# Supplementary material for: Insecticide susceptibility of the sand fly leishmaniasis vector Phlebotomus argentipes in Sri Lanka
Source: Parasit Vectors. 2020 May 13;13:246. doi: 10.1186/s13071-020-04117-y (PMC7218544; doi:10.1186/s13071-020-04117-y)
Supplement: Supplementary file 1 — Additional file 1: Table S1. Statistical analysis to test for significant differences of sand fly mortality between the studied populations to tested insecticides using the Wilcoxon signed-rank test. [file 13071_2020_4117_MOESM1_ESM.pdf]

Additional file 1: Table S1. Statistical analysis to test for significant differences of sand fly mortality between the studied populations to tested insecticides using the Wilcoxon signed-rank test.

Statistical analysis-Bioassays

For DDT insecticide

```
DATASET ACTIVATE DataSet0.
DATASET CLOSE DataSet1.
NPAR TESTS
  /WILCOXON=Mortality_Pannala Mortality_Pannala Mortality_Pannala Mortality_Thalawa
Mortality_Thalawa Mortality_Mirigama WITH Mortality_Thalawa Mortality_Mirigama
Mortality_Mamadala Mortality_Mirigama Mortality_Mamadala Mortality_Mamadala (PAIRED)
  /STATISTICS DESCRIPTIVES
  /MISSING ANALYSIS.
```

NPar Tests

Descriptive Statistics

	N	Mean	Std. Deviation	Minimum	Maximum
Mortality_Pannala	8	86.2500	35.02550	.00	100.00
Mortality_Thalawa	8	85.0000	35.05098	.00	100.00
Mortality_Mirigama	8	86.5000	35.06321	.00	100.00
Mortality_Mamadala	8	80.0000	35.15273	.00	100.00

Wilcoxon Signed Ranks Test

Ranks

		N	Mean Rank	Sum of Ranks
Mortality_Thalawa - Mortality_Pannala	Negative Ranks	1[a]	1.00	1.00
	Positive Ranks	0[b]	.00	.00
	Ties	7[c]		
	Total	8		
Mortality_Mirigama - Mortality_Pannala	Negative Ranks	0[d]	.00	.00
	Positive Ranks	1[e]	1.00	1.00
	Ties	7[f]		
	Total	8		
Mortality_Mamadala - Mortality_Pannala	Negative Ranks	2[g]	1.50	3.00
	Positive Ranks	0[h]	.00	.00
	Ties	6[i]		

		N	Mean Rank	Sum of Ranks
	Total	8		
Mortality_Mirigama - Mortality_Thalawa	Negative Ranks	0[j]	.00	.00
	Positive Ranks	1[k]	1.00	1.00
	Ties	7[l]		
	Total	8		
Mortality_Mamadala - Mortality_Thalawa	Negative Ranks	2[m]	1.50	3.00
	Positive Ranks	0[n]	.00	.00
	Ties	6[o]		
	Total	8		
Mortality_Mamadala - Mortality_Mirigama	Negative Ranks	2[p]	1.50	3.00
	Positive Ranks	0[q]	.00	.00
	Ties	6[r]		
	Total	8		

a. Mortality_Thalawa < Mortality_Pannala
b. Mortality_Thalawa > Mortality_Pannala
c. Mortality_Thalawa = Mortality_Pannala
d. Mortality_Mirigama < Mortality_Pannala
e. Mortality_Mirigama > Mortality_Pannala
f. Mortality_Mirigama = Mortality_Pannala
g. Mortality_Mamadala < Mortality_Pannala
h. Mortality_Mamadala > Mortality_Pannala
i. Mortality_Mamadala = Mortality_Pannala
j. Mortality_Mirigama < Mortality_Thalawa
k. Mortality_Mirigama > Mortality_Thalawa
l. Mortality_Mirigama = Mortality_Thalawa
m. Mortality_Mamadala < Mortality_Thalawa
n. Mortality_Mamadala > Mortality_Thalawa
o. Mortality_Mamadala = Mortality_Thalawa
p. Mortality_Mamadala < Mortality_Mirigama
q. Mortality_Mamadala > Mortality_Mirigama
r. Mortality_Mamadala = Mortality_Mirigama

Test Statistics[a]

	Mortality_Thalawa - Mortality_Pannala	Mortality_Mirigama - Mortality_Pannala	Mortality_Mamadala - Mortality_Pannala	Mortality_Mirigama - Mortality_Thalawa	Mortality_Mamadala - Mortality_Thalawa	Mortality_Mamadala - Mortality_Mirigama
Z	-1.000[b]	-1.000[c]	-1.414[b]	-1.000[c]	-1.342[b]	-1.342[b]
Asymp. Sig. (2-tailed)	.317	.317	.157	.317	.180	.180

a. Wilcoxon Signed Ranks Test
b. Based on positive ranks.
c. Based on negative ranks.

Significant values were >0.05 (0.157-0.317) for DDT insecticide. Therefore, there is no significant difference among these four populations.

For malathion insecticide

```
NPAR TESTS
  /WILCOXON=Mortality_Mamadala Mortality_Mamadala Mortality_Mamadala Mortality_Thalawa
Mortality_Thalawa Mortality_Pannala WITH Mortality_Thalawa Mortality_Pannala
Mortality_Mirigama Mortality_Pannala Mortality_Mirigama Mortality_Mirigama (PAIRED)
  /STATISTICS DESCRIPTIVES
  /MISSING ANALYSIS.
```

NPar Tests

```
[DataSet0]
```

Descriptive Statistics

	N	Mean	Std. Deviation	Minimum	Maximum
Mortality_Mamadala	8	80.0000	35.25013	.00	100.00
Mortality_Thalawa	8	84.7500	35.09884	.00	100.00
Mortality_Pannala	8	86.0000	35.00204	.00	100.00
Mortality_Mirigama	8	86.2500	35.02550	.00	100.00

Wilcoxon Signed Ranks Test

Ranks

		N	Mean Rank	Sum of Ranks
Mortality_Thalawa - Mortality_Mamadala	Negative Ranks	0[a]	.00	.00
	Positive Ranks	2[b]	1.50	3.00
	Ties	6[c]		
	Total	8		
Mortality_Pannala - Mortality_Mamadala	Negative Ranks	0[d]	.00	.00
	Positive Ranks	2[e]	1.50	3.00
	Ties	6[f]		
	Total	8		
Mortality_Mirigama - Mortality_Mamadala	Negative Ranks	0[g]	.00	.00
	Positive Ranks	2[h]	1.50	3.00
	Ties	6[i]		
	Total	8		
Mortality_Pannala - Mortality_Thalawa	Negative Ranks	0[j]	.00	.00
	Positive Ranks	1[k]	1.00	1.00
	Ties	7[l]		
	Total	8		
Mortality_Mirigama - Mortality_Thalawa	Negative Ranks	0[m]	.00	.00

	Positive Ranks	1[n]	1.00	1.00
	Ties	7[o]		
	Total	8		
Mortality_Mirigama - Mortality_Pannala	Negative Ranks	0[p]	.00	.00
	Positive Ranks	1[q]	1.00	1.00
	Ties	7[r]		
	Total	8		

a. Mortality_Thalawa < Mortality_Mamadala

b. Mortality_Thalawa > Mortality_Mamadala

c. Mortality_Thalawa = Mortality_Mamadala

d. Mortality_Pannala < Mortality_Mamadala

e. Mortality_Pannala > Mortality_Mamadala

f. Mortality_Pannala = Mortality_Mamadala

g. Mortality_Mirigama < Mortality_Mamadala

h. Mortality_Mirigama > Mortality_Mamadala

i. Mortality_Mirigama = Mortality_Mamadala

j. Mortality_Pannala < Mortality_Thalawa

k. Mortality_Pannala > Mortality_Thalawa

l. Mortality_Pannala = Mortality_Thalawa

m. Mortality_Mirigama < Mortality_Thalawa

n. Mortality_Mirigama > Mortality_Thalawa

o. Mortality_Mirigama = Mortality_Thalawa

p. Mortality_Mirigama < Mortality_Pannala

q. Mortality_Mirigama > Mortality_Pannala

r. Mortality_Mirigama = Mortality_Pannala

Test Statistics[a]

	Mortality_Thalawa - Mortality_Mamadala	Mortality_Pannala - Mortality_Mamadala	Mortality_Mirigama - Mortality_Mamadala	Mortality_Pannala - Mortality_Thalawa	Mortality_Mirigama - Mortality_Thalawa	Mortality_Mirigama - Mortality_Pannala
Z	-1.342[b]	-1.342[b]	-1.342[b]	-1.000[b]	-1.000[b]	-1.000[b]
Asymp. Sig. (2-tailed)	.180	.180	.180	.317	.317	.317

a. Wilcoxon Signed Ranks Test

b. Based on negative ranks.

Significant values were >0.05 (0.180-0.317) for malathion insecticide. Therefore, there is no significant difference among these four populations.

For propoxur insecticide

```
NEW FILE.
DATASET NAME DataSet1 WINDOW=FRONT.
NPAR TESTS
  /WILCOXON=Mortality_Mamadala Mortality_Mamadala Mortality_Mamadala Mortality_Thalawa
Mortality_Thalawa Mortality_Pannala WITH Mortality_Thalawa Mortality_Pannala
Mortality_Mirigama Mortality_Pannala Mortality_Mirigama Mortality_Mirigama (PAIRED)
  /STATISTICS DESCRIPTIVES
  /MISSING ANALYSIS.
```

NPar Tests

[DataSet1]

Descriptive Statistics

	N	Mean	Std. Deviation	Minimum	Maximum
Mortality_Mamadala	5	68.4000	40.99146	.00	100.00
Mortality_Thalawa	5	76.4000	43.41428	.00	100.00
Mortality_Pannala	5	78.0000	43.81780	.00	100.00
Mortality_Mirigama	5	78.4000	43.96362	.00	100.00

Wilcoxon Signed Ranks Test

Ranks

		N	Mean Rank	Sum of Ranks
Mortality_Thalawa - Mortality_Mamadala	Negative Ranks	0[a]	.00	.00
	Positive Ranks	2[b]	1.50	3.00
	Ties	3[c]		
	Total	5		
Mortality_Pannala - Mortality_Mamadala	Negative Ranks	0[d]	.00	.00
	Positive Ranks	2[e]	1.50	3.00
	Ties	3[f]		
	Total	5		
Mortality_Mirigama - Mortality_Mamadala	Negative Ranks	0[g]	.00	.00
	Positive Ranks	2[h]	1.50	3.00
	Ties	3[i]		
	Total	5		
Mortality_Pannala - Mortality_Thalawa	Negative Ranks	0[j]	.00	.00
	Positive Ranks	1[k]	1.00	1.00
	Ties	4[l]		
	Total	5		
Mortality_Mirigama - Mortality_Thalawa	Negative Ranks	0[m]	.00	.00
	Positive Ranks	1[n]	1.00	1.00
	Ties	4[o]		
	Total	5		
Mortality_Mirigama - Mortality_Pannala	Negative Ranks	0[p]	.00	.00
	Positive Ranks	1[q]	1.00	1.00
	Ties	4[r]		
	Total	5		

a. Mortality_Thalawa < Mortality_Mamadala

b. Mortality_Thalawa > Mortality_Mamadala

c. Mortality_Thalawa = Mortality_Mamadala

d. Mortality_Pannala < Mortality_Mamadala

e. Mortality_Pannala > Mortality_Mamadala

f. Mortality_Pannala = Mortality_Mamadala

g. Mortality_Mirigama < Mortality_Mamadala

h. Mortality_Mirigama > Mortality_Mamadala

i. Mortality_Mirigama = Mortality_Mamadala

j. Mortality_Pannala < Mortality_Thalawa

k. Mortality_Pannala > Mortality_Thalawa

l. Mortality_Pannala = Mortality_Thalawa

m. Mortality_Mirigama < Mortality_Thalawa

n. Mortality_Mirigama > Mortality_Thalawa

o. Mortality_Mirigama = Mortality_Thalawa

p. Mortality_Mirigama < Mortality_Pannala

q. Mortality_Mirigama > Mortality_Pannala

r. Mortality_Mirigama = Mortality_Pannala

Test Statistics[a]

	Mortality_Thalawa - Mortality_Mamadala	Mortality_Pannala - Mortality_Mamadala	Mortality_Mirigama - Mortality_Mamadala	Mortality_Pannala - Mortality_Thalawa	Mortality_Mirigama - Mortality_Thalawa	Mortality_Mirigama - Mortality_Pannala
Z	-1.342[b]	-1.342[b]	-1.414[b]	-1.000[b]	-1.000[b]	-1.000[b]
Asymp. Sig. (2-tailed)	.180	.180	.157	.317	.317	.317

a. Wilcoxon Signed Ranks Test

b. Based on negative ranks.

Significant values were >0.05 (0.157-0.317) for propoxur insecticide. Therefore, there is no significant difference among these four populations.

For deltamethrin insecticide

```
NEW FILE.
DATASET NAME DataSet2 WINDOW=FRONT.
NPAR TESTS
  /WILCOXON=Mortality_Mamadala Mortality_Mamadala Mortality_Mamadala Mortality_Thalawa
Mortality_Thalawa Mortality_Pannala WITH Mortality_Thalawa Mortality_Pannala
Mortality_Mirigama Mortality_Pannala Mortality_Mirigama Mortality_Mirigama (PAIRED)
  /STATISTICS DESCRIPTIVES
  /MISSING ANALYSIS.
```

NPar Tests

```
[DataSet2]
```

Descriptive Statistics

	N	Mean	Std. Deviation	Minimum	Maximum
Mortality_Mamadala	8	87.5000	35.35534	.00	100.00
Mortality_Thalawa	8	87.5000	35.35534	.00	100.00
Mortality_Pannala	8	87.5000	35.35534	.00	100.00
Mortality_Mirigama	8	91.2500	24.74874	30.00	100.00

Wilcoxon Signed Ranks Test

Ranks

		N	Mean Rank	Sum of Ranks
Mortality_Thalawa - Mortality_Mamadala	Negative Ranks	0[a]	.00	.00
	Positive Ranks	0[b]	.00	.00
	Ties	8[c]		
	Total	8		
Mortality_Pannala - Mortality_Mamadala	Negative Ranks	0[d]	.00	.00
	Positive Ranks	0[e]	.00	.00
	Ties	8[f]		
	Total	8		
Mortality_Mirigama - Mortality_Mamadala	Negative Ranks	0[g]	.00	.00
	Positive Ranks	1[h]	1.00	1.00
	Ties	7[i]		
	Total	8		
Mortality_Pannala - Mortality_Thalawa	Negative Ranks	0[j]	.00	.00
	Positive Ranks	0[k]	.00	.00
	Ties	8[l]		
	Total	8		
Mortality_Mirigama - Mortality_Thalawa	Negative Ranks	0[m]	.00	.00
	Positive Ranks	1[n]	1.00	1.00
	Ties	7[o]		
	Total	8		
Mortality_Mirigama - Mortality_Pannala	Negative Ranks	0[p]	.00	.00
	Positive Ranks	1[q]	1.00	1.00
	Ties	7[r]		
	Total	8		

a. Mortality_Thalawa < Mortality_Mamadala

b. Mortality_Thalawa > Mortality_Mamadala

c. Mortality_Thalawa = Mortality_Mamadala

d. Mortality_Pannala < Mortality_Mamadala

e. Mortality_Pannala > Mortality_Mamadala

f. Mortality_Pannala = Mortality_Mamadala

g. Mortality_Mirigama < Mortality_Mamadala

h. Mortality_Mirigama > Mortality_Mamadala

i. Mortality_Mirigama = Mortality_Mamadala

j. Mortality_Pannala < Mortality_Thalawa

k. Mortality_Pannala > Mortality_Thalawa

l. Mortality_Pannala = Mortality_Thalawa

m. Mortality_Mirigama < Mortality_Thalawa

n. Mortality_Mirigama > Mortality_Thalawa

o. Mortality_Mirigama = Mortality_Thalawa

p. Mortality_Mirigama < Mortality_Pannala

q. Mortality_Mirigama > Mortality_Pannala

r. Mortality_Mirigama = Mortality_Pannala

Test Statistics[a]

	Mortality_Thalawa - Mortality_Mamadala	Mortality_Pannala - Mortality_Mamadala	Mortality_Mirigama - Mortality_Mamadala	Mortality_Pannala - Mortality_Thalawa	Mortality_Mirigama - Mortality_Thalawa	Mortality_Mirigama - Mortality_Pannala
Z	.000[b]	.000[b]	-1.000[c]	.000[b]	-1.000[c]	-1.000[c]
Asymp. Sig. (2-tailed)	1.000	1.000	.317	1.000	.317	.317

a. Wilcoxon Signed Ranks Test

b. The sum of negative ranks equals the sum of positive ranks.

c. Based on negative ranks.

Significant values were >0.05 (0.317-1.000) for deltamethrin insecticide. Therefore, there is no significant difference among these four populations.

The 95% confidence interval limits (lower and upper) were calculated using the 1-sample t (Test and confidence level) in Minitab (version 15) for DDT, malathion, propoxur and deltamethrin in each study site.

DDT

Pannala

Variable	N	Mean	StDev	SE Mean	95% CI
0.4	5	0.000000	0.000000	0.000000	(0.000000, 0.000000)=0
0.6	5	0.000000	0.000000	0.000000	(0.000000, 0.000000)=0
0.8	5	0.000000	0.000000	0.000000	(0.000000, 0.000000)=0
1.5	5	61.0000	1.5811	0.7071	(59.0368, 62.9632)=1.9632
2.0	5	61.0000	1.5811	0.7071	(59.0368, 62.9632)=1.9632
2.5	5	61.8000	1.9235	0.8602	(59.4116, 64.1884)=2.3884
3.0	5	63.0000	1.5811	0.7071	(61.0368, 64.9632)=1.9632
4.0	5	64.0000	1.5811	0.7071	(62.0368, 65.9632)=1.9632
0.4	5	0.000000	0.000000	0.000000	(0.000000, 0.000000)=0
0.6	5	90.0000	1.5811	0.7071	(88.0368, 91.9632)=1.9632
0.8	5	100.000	0.000	0.000	(100.000, 100.000)=0
1.5	5	100.000	0.000	0.000	(100.000, 100.000)=0
2.0	5	100.000	0.000	0.000	(100.000, 100.000)=0
2.5	5	100.000	0.000	0.000	(100.000, 100.000)=0
3.0	5	100.000	0.000	0.000	(100.000, 100.000)=0
4.0	5	100.000	0.000	0.000	(100.000, 100.000)=0

Thalawa

Variable	N	Mean	StDev	SE Mean	95% CI
0.4	5	0.000000	0.000000	0.000000	(0.000000, 0.000000)=0
0.6	5	0.000000	0.000000	0.000000	(0.000000, 0.000000)=0
0.8	5	0.000000	0.000000	0.000000	(0.000000, 0.000000)=0
1.5	5	56.0000	1.5811	0.7071	(54.0368, 57.9632)=1.9632
2.0	5	57.2000	2.5884	1.1576	(53.9860, 60.4140)=3.214
2.5	5	60.0000	1.5811	0.7071	(58.0368, 61.9632)=1.9632
3.0	5	62.0000	1.5811	0.7071	(60.0368, 63.9632)=1.9632
4.0	5	66.0000	1.5811	0.7071	(64.0368, 67.9632)=1.9632
0.4	5	0.000000	0.000000	0.000000	(0.000000, 0.000000)=0
0.6	5	80.0000	1.5811	0.7071	(78.0368, 81.9632)=1.9632
0.8	5	100.000	0.000	0.000	(100.000, 100.000)=0
1.5	5	100.000	0.000	0.000	(100.000, 100.000)=0
2.0	5	100.000	0.000	0.000	(100.000, 100.000)=0
2.5	5	100.000	0.000	0.000	(100.000, 100.000)=0
3.0	5	100.000	0.000	0.000	(100.000, 100.000)=0
4.0	5	100.000	0.000	0.000	(100.000, 100.000)=0

Mamadala

Variable	N	Mean	StDev	SE Mean	95% CI
0.4	5	0.000000	0.000000	0.000000	(0.000000, 0.000000)=0
0.6	5	0.000000	0.000000	0.000000	(0.000000, 0.000000)=0
0.8	5	0.000000	0.000000	0.000000	(0.000000, 0.000000)=0
1.5	5	55.0000	1.5811	0.7071	(53.0368, 56.9632)=1.9632
2.0	5	58.0000	1.5811	0.7071	(56.0368, 59.9632)=1.9632
2.5	5	60.2000	1.3038	0.5831	(58.5811, 61.8189)=1.6189
3.0	5	62.4000	1.8166	0.8124	(60.1444, 64.6556)=2.2556
4.0	5	62.4000	1.8166	0.8124	(60.1444, 64.6556)=2.2556
0.4	5	0.000000	0.000000	0.000000	(0.000000, 0.000000)=0
0.6	5	65.4000	1.5166	0.6782	(63.5169, 67.2831)=1.8831
0.8	5	75.2000	0.8367	0.3742	(74.1611, 76.2389)=1.0389
1.5	5	100.000	0.000	0.000	(100.000, 100.000)=0
2.0	5	100.000	0.000	0.000	(100.000, 100.000)=0
2.5	5	100.000	0.000	0.000	(100.000, 100.000)=0
3.0	5	100.000	0.000	0.000	(100.000, 100.000)=0
4.0	5	100.000	0.000	0.000	(100.000, 100.000)=0

Mirigama

Variable	N	Mean	StDev	SE Mean	95% CI
0.4	5	0.000000	0.000000	0.000000	(0.000000, 0.000000)=0
0.6	5	0.000000	0.000000	0.000000	(0.000000, 0.000000)=0
0.8	5	40.8000	1.3038	0.5831	(39.1811, 42.4189)=1.6189
1.5	5	47.4000	1.1402	0.5099	(45.9843, 48.8157)=1.4157
2.0	5	50.6000	1.1402	0.5099	(49.1843, 52.0157)=1.4157
2.5	5	52.4000	1.1402	0.5099	(50.9843, 53.8157)=1.4157
3.0	5	53.0000	1.5811	0.7071	(51.0368, 54.9632)=1.9632
4.0	5	56.0000	1.5811	0.7071	(54.0368, 57.9632)=1.9632
0.4	5	0.000000	0.000000	0.000000	(0.000000, 0.000000)=0
0.6	5	91.0000	1.5811	0.7071	(89.0368, 92.9632)=1.9632
0.8	5	100.000	0.000	0.000	(100.000, 100.000)=0
1.5	5	100.000	0.000	0.000	(100.000, 100.000)=0
2.0	5	100.000	0.000	0.000	(100.000, 100.000)=0
2.5	5	100.000	0.000	0.000	(100.000, 100.000)=0
3.0	5	100.000	0.000	0.000	(100.000, 100.000)=0
4.0	5	100.000	0.000	0.000	(100.000, 100.000)=0

Malathion

Pannala

Variable	N	Mean	StDev	SE Mean	95% CI
0.5	5	0.000000	0.000000	0.000000	(0.000000, 0.000000)=0
0.7	5	0.000000	0.000000	0.000000	(0.000000, 0.000000)=0
0.9	5	0.000000	0.000000	0.000000	(0.000000, 0.000000)=0
2.0	5	67.0000	1.5811	0.7071	(65.0368, 68.9632)=1.9632
2.5	5	74.0000	1.5811	0.7071	(72.0368, 75.9632)=1.9632
3.0	5	74.0000	1.5811	0.7071	(72.0368, 75.9632)=1.9632
3.5	5	75.0000	1.5811	0.7071	(73.0368, 76.9632)=1.9632
5.0	5	76.0000	1.5811	0.7071	(74.0368, 77.9632)=1.9632
0.5	5	0.000000	0.000000	0.000000	(0.000000, 0.000000)=0
0.7	5	87.6000	1.1402	0.5099	(86.1843, 89.0157)=1.4157
0.9	5	100.000	0.000	0.000	(100.000, 100.000)=0
2.0	5	100.000	0.000	0.000	(100.000, 100.000)=0
2.5	5	100.000	0.000	0.000	(100.000, 100.000)=0
3.0	5	100.000	0.000	0.000	(100.000, 100.000)=0
3.5	5	100.000	0.000	0.000	(100.000, 100.000)=0
5.0	5	100.000	0.000	0.000	(100.000, 100.000)=0

Thalawa

Variable	N	Mean	StDev	SE Mean	95% CI
0.5	5	0.000000	0.000000	0.000000	(0.000000, 0.000000)=0
0.7	5	0.000000	0.000000	0.000000	(0.000000, 0.000000)=0
0.9	5	0.000000	0.000000	0.000000	(0.000000, 0.000000)=0
2.0	5	65.8000	1.3038	0.5831	(64.1811, 67.4189)=1.6185
2.5	5	71.2000	1.3038	0.5831	(69.5811, 72.8189)=1.6189
3.0	5	72.6000	1.1402	0.5099	(71.1843, 74.0157)=1.4157
3.5	5	73.2000	0.8367	0.3742	(72.1611, 74.2389)=1.0389
5.0	5	74.2000	1.3038	0.5831	(72.5811, 75.8189)=1.6189
0.5	5	0.000000	0.000000	0.000000	(0.000000, 0.000000)=0
0.7	5	78.0000	1.0000	0.4472	(76.7583, 79.2417)=1.2147
0.9	5	100.000	0.000	0.000	(100.000, 100.000)=0
2.0	5	100.000	0.000	0.000	(100.000, 100.000)=0
2.5	5	100.000	0.000	0.000	(100.000, 100.000)=0
3.0	5	100.000	0.000	0.000	(100.000, 100.000)=0
3.5	5	100.000	0.000	0.000	(100.000, 100.000)=0
5.0	5	100.000	0.000	0.000	(100.000, 100.000)=0

Mamadala

Variable	N	Mean	StDev	SE Mean	95% CI
0.5	5	0.000000	0.000000	0.000000	(0.000000, 0.000000)=0
0.7	5	0.000000	0.000000	0.000000	(0.000000, 0.000000)=0
0.9	5	0.000000	0.000000	0.000000	(0.000000, 0.000000)=0
2.0	5	58.8000	1.3038	0.5831	(57.1811, 60.4189)=1.6189
2.5	5	62.4000	1.1402	0.5099	(60.9843, 63.8157)=1.4157
3.0	5	63.6000	1.1402	0.5099	(62.1843, 65.0157)=1.4157
3.5	5	64.8000	1.3038	0.5831	(63.1811, 66.4189)=1.6189
5.0	5	67.2000	1.4832	0.6633	(65.3583, 69.0417)=1.8417
0.5	5	0.000000	0.000000	0.000000	(0.000000, 0.000000)=0
0.7	5	61.8000	1.3038	0.5831	(60.1811, 63.4189)=1.6189
0.9	5	76.6000	1.1402	0.5099	(75.1843, 78.0157)=1.4157
2.0	5	100.000	0.000	0.000	(100.000, 100.000)=0
2.5	5	100.000	0.000	0.000	(100.000, 100.000)=0
3.0	5	100.000	0.000	0.000	(100.000, 100.000)=0
3.5	5	100.000	0.000	0.000	(100.000, 100.000)=0
5.0	5	100.000	0.000	0.000	(100.000, 100.000)=0

Mirigama

Variable	N	Mean	StDev	SE Mean	95% CI
0.5	5	0.000000	0.000000	0.000000	(0.000000, 0.000000)=0
0.7	5	0.000000	0.000000	0.000000	(0.000000, 0.000000)=0
0.9	5	42.2000	0.8367	0.3742	(41.1611, 43.2389)=1.0389
2.0	5	44.6000	1.1402	0.5099	(43.1843, 46.0157)=1.4157
2.5	5	48.2000	0.8367	0.3742	(47.1611, 49.2389)=1.0389
3.0	5	50.4000	1.1402	0.5099	(48.9843, 51.8157)=1.4157
3.5	5	52.4000	1.1402	0.5099	(50.9843, 53.8157)=1.4157
5.0	5	54.2000	0.8367	0.3742	(53.1611, 55.2389)=1.0389
0.5	5	0.000000	0.000000	0.000000	(0.000000, 0.000000)=0
0.7	5	90.4000	1.1402	0.5099	(88.9843, 91.8157)=1.4157
0.9	5	100.000	0.000	0.000	(100.000, 100.000)=0
2.0	5	100.000	0.000	0.000	(100.000, 100.000)=0
2.5	5	100.000	0.000	0.000	(100.000, 100.000)=0
3.0	5	100.000	0.000	0.000	(100.000, 100.000)=0
3.5	5	100.000	0.000	0.000	(100.000, 100.000)=0
5.0	5	100.000	0.000	0.000	(100.000, 100.000)=0

Propoxur

Pannala

Variable	N	Mean	StDev	SE Mean	95% CI
0.01	5	0.000000	0.000000	0.000000	(0.000000, 0.000000)=0
0.015	5	0.000000	0.000000	0.000000	(0.000000, 0.000000)=0
0.017	5	0.000000	0.000000	0.000000	(0.000000, 0.000000)=0
0.03	5	62.6000	1.1402	0.5099	(61.1843, 64.0157)=1.4157
0.07	5	64.0000	1.5811	0.7071	(62.0368, 65.9632)=1.9632
0.1	5	64.0000	1.5811	0.7071	(62.0368, 65.9632)=1.9632
0.01	5	0.000000	0.000000	0.000000	(0.000000, 0.000000)=0
0.015	5	90.0000	1.5811	0.7071	(88.0368, 91.9632)=1.9632
0.017	5	100.000	0.000	0.000	(100.000, 100.000)=0
0.03	5	100.000	0.000	0.000	(100.000, 100.000)=0
0.07	5	100.000	0.000	0.000	(100.000, 100.000)=0
0.1	5	100.000	0.000	0.000	(100.000, 100.000)=0

Thalawa

Variable	N	Mean	StDev	SE Mean	95% CI
0.01	5	0.000000	0.000000	0.000000	(0.000000, 0.000000)=0
0.015	5	0.000000	0.000000	0.000000	(0.000000, 0.000000)=0
0.017	5	0.000000	0.000000	0.000000	(0.000000, 0.000000)=0
0.03	5	60.0000	1.5811	0.7071	(58.0368, 61.9632)=1.9632
0.07	5	63.6000	1.1402	0.5099	(62.1843, 65.0157)=1.4157
0.1	5	64.0000	1.5811	0.7071	(62.0368, 65.9632)=1.9632
0.01	5	0.000000	0.000000	0.000000	(0.000000, 0.000000)=0
0.015	5	82.0000	1.5811	0.7071	(80.0368, 83.9632)=1.9632
0.017	5	100.000	0.000	0.000	(100.000, 100.000)=0
0.03	5	100.000	0.000	0.000	(100.000, 100.000)=0
0.07	5	100.000	0.000	0.000	(100.000, 100.000)=0
0.1	5	100.000	0.000	0.000	(100.000, 100.000)=0

Mamadala

Variable	N	Mean	StDev	SE Mean	95% CI
0.01	5	0.000000	0.000000	0.000000	(0.000000, 0.000000)=0
0.015	5	0.000000	0.000000	0.000000	(0.000000, 0.000000)=0
0.017	5	0.000000	0.000000	0.000000	(0.000000, 0.000000)=0
0.03	5	55.2000	1.3038	0.5831	(53.5811, 56.8189)=1.6189
0.07	5	56.8000	1.3038	0.5831	(55.1811, 58.4189)=1.6189
0.1	5	57.8000	0.8367	0.3742	(56.7611, 58.8389)=1.0389
0.01	5	0.000000	0.000000	0.000000	(0.000000, 0.000000)=0
0.015	5	67.4000	1.1402	0.5099	(65.9843, 68.8157)=1.4157
0.017	5	76.0000	1.5811	0.7071	(74.0368, 77.9632)=1.9632
0.03	5	100.000	0.000	0.000	(100.000, 100.000)=0
0.07	5	100.000	0.000	0.000	(100.000, 100.000)=0
0.1	5	100.000	0.000	0.000	(100.000, 100.000)=0

Mirigama

Variable	N	Mean	StDev	SE Mean	95% CI
0.01	5	0.000000	0.000000	0.000000	(0.000000, 0.000000)=0
0.015	5	0.000000	0.000000	0.000000	(0.000000, 0.000000)=0
0.017	5	30.4000	1.1402	0.5099	(28.9843, 31.8157)=1.4157
0.03	5	40.0000	1.5811	0.7071	(38.0368, 41.9632)=1.9632
0.07	5	51.0000	1.5811	0.7071	(49.0368, 52.9632)=1.9632
0.1	5	60.0000	1.5811	0.7071	(58.0368, 61.9632)=1.9632
0.01	5	0.000000	0.000000	0.000000	(0.000000, 0.000000)=0
0.015	5	91.0000	1.5811	0.7071	(89.0368, 92.9632)=1.9632
0.017	5	100.000	0.000	0.000	(100.000, 100.000)=0
0.03	5	100.000	0.000	0.000	(100.000, 100.000)=0
0.07	5	100.000	0.000	0.000	(100.000, 100.000)=0
0.1	5	100.000	0.000	0.000	(100.000, 100.000)=0

Deltamethrin

Pannala

Variable	N	Mean	StDev	SE Mean	95% CI
0.005	5	0.000000	0.000000	0.000000	(0.000000, 0.000000)=0
0.007	5	42.4000	1.1402	0.5099	(40.9843, 43.8157)=1.4157
0.009	5	51.4000	1.1402	0.5099	(49.9843, 52.8157)=1.4157
0.02	5	64.0000	1.5811	0.7071	(62.0368, 65.9632)=1.9632
0.025	5	64.0000	1.5811	0.7071	(62.0368, 65.9632)=1.9632
0.03	5	65.0000	1.5811	0.7071	(63.0368, 66.9632)=0.9816
0.035	5	67.0000	1.5811	0.7071	(65.0368, 68.9632)=1.9632
0.05	5	67.0000	1.5811	0.7071	(65.0368, 68.9632)=1.9632
0.005	5	0.000000	0.000000	0.000000	(0.000000, 0.000000)=0
0.007	5	100.000	0.000	0.000	(100.000, 100.000)=0
0.009	5	100.000	0.000	0.000	(100.000, 100.000)=0
0.02	5	100.000	0.000	0.000	(100.000, 100.000)=0
0.025	5	100.000	0.000	0.000	(100.000, 100.000)=0

0.03	5	100.000	0.000	0.000	(100.000, 100.000)=0
0.035	5	100.000	0.000	0.000	(100.000, 100.000)=0
0.05	5	100.000	0.000	0.000	(100.000, 100.000)=0

Thalawa

Variable	N	Mean	StDev	SE Mean	95% CI
0.005	5	0.000000	0.000000	0.000000	(0.000000, 0.000000)=0
0.007	5	40.0000	1.5811	0.7071	(38.0368, 41.9632)=1.9632
0.009	5	47.0000	1.5811	0.7071	(45.0368, 48.9632)=1.9632
0.02	5	54.0000	1.5811	0.7071	(52.0368, 55.9632)=1.9632
0.025	5	57.6000	1.1402	0.5099	(56.1843, 59.0157)=1.4157
p0.03	5	60.2000	1.9235	0.8602	(57.8116, 62.5884)=2.3884
0.035	5	66.0000	1.5811	0.7071	(64.0368, 67.9632)=1.9632
0.05	5	66.0000	1.5811	0.7071	(64.0368, 67.9632)=1.9632
0.005	5	0.000000	0.000000	0.000000	(0.000000, 0.000000)=0
0.007	5	100.000	0.000	0.000	(100.000, 100.000)=0
0.009	5	100.000	0.000	0.000	(100.000, 100.000)=0
0.02	5	100.000	0.000	0.000	(100.000, 100.000)=0
0.025	5	100.000	0.000	0.000	(100.000, 100.000)=0
0.03	5	100.000	0.000	0.000	(100.000, 100.000)=0
0.035	5	100.000	0.000	0.000	(100.000, 100.000)=0
0.05	5	100.000	0.000	0.000	(100.000, 100.000)=0

Mamadala

Variable	N	Mean	StDev	SE Mean	95% CI
0.005	5	0.000000	0.000000	0.000000	(0.000000, 0.000000)=0
0.007	5	34.0000	1.5811	0.7071	(32.0368, 35.9632)=1.9632
0.009	5	42.2000	1.3038	0.5831	(40.5811, 43.8189)=1.6189
0.02	5	53.0000	1.5811	0.7071	(51.0368, 54.9632)=1.9632
0.025	5	54.8000	1.9235	0.8602	(52.4116, 57.1884)=2.3884
p0.03	5	55.4000	1.5166	0.6782	(53.5169, 57.2831)=1.8831
0.035	5	57.0000	1.5811	0.7071	(55.0368, 58.9632)=1.9632
0.05	5	57.0000	1.5811	0.7071	(55.0368, 58.9632)=1.9632
0.005	5	0.000000	0.000000	0.000000	(0.000000, 0.000000)=0
0.007	5	100.000	0.000	0.000	(100.000, 100.000)=0
0.009	5	100.000	0.000	0.000	(100.000, 100.000)=0
0.02	5	100.000	0.000	0.000	(100.000, 100.000)=0
0.025	5	100.000	0.000	0.000	(100.000, 100.000)=0
0.03	5	100.000	0.000	0.000	(100.000, 100.000)=0
0.035	5	100.000	0.000	0.000	(100.000, 100.000)=0
0.05	5	100.000	0.000	0.000	(100.000, 100.000)=0

Mirigama

Variable	N	Mean	StDev	SE Mean	95% CI
0.005	5	0.000000	0.000000	0.000000	(0.000000, 0.000000)=0
0.007	5	20.0000	1.5811	0.7071	(18.0368, 21.9632)=1.9632
0.009	5	34.0000	1.5811	0.7071	(32.0368, 35.9632)=1.9632
0.02	5	38.0000	1.5811	0.7071	(36.0368, 39.9632)=1.9632
0.025	5	45.2000	1.9235	0.8602	(42.8116, 47.5884)=2.3884
0.03	5	52.4000	2.0736	0.9274	(49.8252, 54.9748)=2.5748
0.035	5	60.0000	1.5811	0.7071	(58.0368, 61.9632)=1.9632
0.05	5	67.2000	1.9235	0.8602	(64.8116, 69.5884)=2.3884
0.005	5	30.4000	2.0736	0.9274	(27.8252, 32.9748)=2.5748
0.007	5	100.000	0.000	0.000	(100.000, 100.000)=0
0.009	5	100.000	0.000	0.000	(100.000, 100.000)=0
0.02	5	100.000	0.000	0.000	(100.000, 100.000)=0
0.025	5	100.000	0.000	0.000	(100.000, 100.000)=0
p0.03	5	100.000	0.000	0.000	(100.000, 100.000)=0
0.035	5	100.000	0.000	0.000	(100.000, 100.000)=0
0.05	5	100.000	0.000	0.000	(100.000, 100.000)=0
