# Supplementary material for: Insecticide susceptibility of the sand fly leishmaniasis vector Phlebotomus argentipes in Sri Lanka
Source: Parasit Vectors. 2020 May 13;13:246. doi: 10.1186/s13071-020-04117-y (PMC7218544; doi:10.1186/s13071-020-04117-y)
Supplement: Supplementary file 2 — Additional file 2: Text S1. Statistical analysis to test for significant differences using ANOVA of elevated enzymes and remaining enzyme activities in each location. [file 13071_2020_4117_MOESM2_ESM.pdf]

Additional file 2: Text S1. Statistical analysis to test for significant differences using ANOVA of elevated enzymes and remaining enzyme activities in each location.

Statistical analyses- Biochemical analysis

Descriptive statistics in Minitab (version 15) were used to test mean values and standard errors of each assays in each populations (Table 2).

One-way ANOVA was used to compare the acetylcholinesterase remaining activity (Ache), esterase (Est), glutathione-s-transferase (Gst) and monooxygenases activities in four populations (Table 2).

One-way ANOVA: Ache_Mamadala, Ache_Thalawa, Ache_Pannala, Ache_Mirigama

```
Source  DF   SS  MS   F   P
Factor  3  19412 6471 14.75 0.000
Error  792 347414  439
Total  795 366826

S = 20.94  R-Sq = 5.29%  R-Sq(adj) = 4.93%


                 Individual 95% CIs For Mean Based on Pooled
                 StDev
Level      N  Mean StDev   +---------+---------+---------+---------
Ache_Mamadala 200 24.62 23.84              (-----*-----)
Ache_Thalawa  200 24.06 24.00              (-----*-----)
Ache_Pannala  200 23.50 21.70             (-----*-----)
Ache_Mirigama 200 12.69 11.80   (----*-----)
                   +---------+---------+---------+---------
                 10.0   15.0   20.0   25.0

Pooled StDev = 20.94
```

ANOVA: $F_{(3,792)}$= 14.75, P = 0.000

The remaining activities of AChE target site were significantly higher when compared with that of flies originated from Mirigama (P<0.005).

One-way ANOVA: Ache_Mamadala, Ache_Thalawa, Ache_Pannala

```
Source   DF    SS  MS   F    P
Factor  2   125  63 0.12 0.890
Error  594 319827 538
Total  596 319953

S = 23.20  R-Sq = 0.04%  R-Sq(adj) = 0.00%


                 Individual 95% CIs For Mean Based on
                 Pooled StDev
Level       N  Mean StDev ---------+---------+---------+---------+
Ache_Mamadala 200 24.62 23.84    (---------------*---------------)
Ache_Thalawa  200 24.06 24.00   (---------------*---------------)
Ache_Pannala  200 23.50 21.70 (----------------*----------------)
                 ---------+---------+---------+---------+
                    22.0   24.0   26.0   28.0

Pooled StDev = 23.20
```

ANOVA: $F_{(2, 594)} = 0.12$, $P = 0.890$

The remaining activities of AChE target site were comparable in those flies that originated from Mamadala, Thalawa and Pannala (P>0.05).

One-way ANOVA: Est_Mamadala, Est_Thalawa, Est_Pannala, Est_Mirigama

```
Source   DF       SS      MS     F      P
Factor    3   0.2086  0.0695  5.00  0.002
Error   792  11.0181  0.0139
Total   795  11.2266

S = 0.1179   R-Sq = 1.86%   R-Sq(adj) = 1.49%


                                   Individual 95% CIs For Mean Based on
                                   Pooled StDev
Level           N    Mean   StDev  ----+---------+---------+---------+-----
Est_Mamadala  199  0.1082  0.1315                      (-------*-------)
Est_Thalawa   199  0.1054  0.1306                    (--------*-------)
Est_Pannala   199  0.1055  0.1307                     (-------*-------)
Est_Mirigama  199  0.0690  0.0649  (--------*-------)
                                   ----+---------+---------+---------+-----
                                     0.060     0.080     0.100     0.120

Pooled StDev = 0.1179
```

ANOVA: $F_{(3, 792)} = 5.00$, $P = 0.002$

The mean activities of esterases were significantly higher when compared with that of flies originated from Mirigama (P<0.005).

One-way ANOVA: Est_Mamadala, Est_Thalawa, Est_Pannala

```
Source   DF       SS      MS     F      P
Factor    2   0.0010  0.0005  0.03  0.972
Error   594  10.1834  0.0171
Total   596  10.1844

S = 0.1309   R-Sq = 0.01%   R-Sq(adj) = 0.00%


                                   Individual 95% CIs For Mean Based on
                                   Pooled StDev
Level           N    Mean   StDev  ---+---------+---------+---------+------
Est_Mamadala  199  0.1082  0.1315     (-----------------*-----------------)
```

```
Est_Thalawa   199  0.1054  0.1306  (-----------------*------------------)
Est_Pannala   199  0.1055  0.1307  (-----------------*------------------)
                                   ---+---------+---------+---------+------
                                    0.090     0.100     0.110     0.120

Pooled StDev = 0.1309
```

ANOVA: $F_{(2, 594)} = 0.03$, $P = 0.972$

The mean activities of esterases were comparable in those flies that originated from Mamadala, Thalawa and Pannala (P>0.05).

One-way ANOVA: Gst_Mamadala, Gst_Thalawa, Gst_Pannala, Gst_Mirigama

```
Source   DF       SS      MS     F      P
Factor    3   0.2405  0.0802  2.02  0.110
Error   788  31.3213  0.0397
Total   791  31.5618

S = 0.1994   R-Sq = 0.76%   R-Sq(adj) = 0.38%


                                   Individual 95% CIs For Mean Based on
                                   Pooled StDev
Level           N    Mean   StDev  --+---------+---------+---------+-------
Gst_Mamadala  198  0.1984  0.2036           (--------*--------)
Gst_Thalawa   198  0.2213  0.2333                  (---------*--------)
Gst_Pannala   198  0.1984  0.2036           (--------*--------)
Gst_Mirigama  198  0.1721  0.1472  (--------*---------)
                                   --+---------+---------+---------+-------
                                   0.150     0.180     0.210     0.240

Pooled StDev = 0.1994
```

ANOVA: $F_{(3, 788)} = 2.02$, $P = 0.110$

The mean activities of glutathione-s-transferases were comparable in those flies that originated from Mamadala, Thalawa, Pannala and Mirigama (P>0.05).

One-way ANOVA: Monooxigenas, Monooxigenas, Monooxigenas, Monooxigenas

Source	DF	SS	MS	F	P
Factor	3	0.0002311	0.0000770	2.46	0.062
Error	792	0.0248051	0.0000313		
Total	795	0.0250362			

S = 0.005596   R-Sq = 0.92%   R-Sq(adj) = 0.55%

Level	N	Mean	StDev
Monooxigenases_M	199	0.007374	0.005902
Monooxigenases_T	199	0.006726	0.005877
Monooxigenases_P	199	0.006943	0.006010
Monooxigenases_M	199	0.005893	0.004448

```
                  Individual 95% CIs For Mean Based on
                  Pooled StDev
Level             ------+---------+---------+---------+---
Monooxigenases_M                    (---------*---------)
Monooxigenases_T            (---------*---------)
Monooxigenases_P               (---------*---------)
Monooxigenases_M  (---------*--------)
                  ------+---------+---------+---------+---
                      0.00560   0.00640   0.00720   0.00800
```

Pooled StDev = 0.005596

ANOVA: $F_{(3, 792)} = 2.46$, $P = 0.062$

The mean activities of monooxygenases were comparable in those flies that originated from Mamadala, Thalawa, Pannala and Mirigama (P>0.05).
