# Supplementary material for: Insecticide susceptibility of the sand fly leishmaniasis vector Phlebotomus argentipes in Sri Lanka
Source: Parasit Vectors. 2020 May 13;13:246. doi: 10.1186/s13071-020-04117-y (PMC7218544; doi:10.1186/s13071-020-04117-y)
Supplement: Supplementary file 3 — Additional file 3: Figure S1. Sequence alignment of the VGSC gene in P. argentipes with the sequence for the voltage-sensitive sodium channel of Musca domestica (GenBank: AAB47604). Arrow indicates the position of the L1014F mutation. Heterozygotes samples were indicated with an asterisk (*). [file 13071_2020_4117_MOESM3_ESM.pdf]

Additional file 3: Figure S1. Sequence alignment of the *VGSC* gene in *P. argentipes* with the sequence for the voltage-sensitive sodium channel of *Musca domestica* (GenBank: AAB47604). Arrow indicates the position of the L1014F mutation. Heterozygotes samples were indicated with an asterisk (*).

```
                                 990       1000      1010      1020      1030      1040      1050
                             ....|....|....|....|....|....|.... |....|....|....|....|....|....|....|
AAB47604.1 voltage-s         VLCGEWIESMWDCMYVGDVSCIPFFLATVVIGNL VVLNLFLALLLSNFGSSSLSAPTADNDTNKIAEAFN
K10 3253 aa                  ---------MWDCMLVGDVSCIPFFLATVVIGNF*VVLNLFLALLLSNFGSSSLSAPTADNDT--------
K11 3253 aa                  ---------MWDCMLVGDVSCIPFFLATVVIGNL VVLNLFLA----------------------------
K12 3253 aa                  ---------MWDCMLVGDVSCIPFFLATVVIGNF VVLNLFLALLLSNFGSSSLSAPTADNDT--------
K13 3253 aa                  ---------MWDCMLVGDVSCIPFFLATVVIGNL VVLNLFLALLLSNFGSSSLSAPTADNDT--------
K14 3253 aa                  ---------MWDCMLVGDVSCIPFFLATVVIGNL VVLNLFLALLLSNFGSSSLSAPTADNDT--------
K15 3253 aa                  ---------MWDCMLVGDVSCIPFFLATVVIGNF VVLNLFLALLLSNFGSSSLSAPTADNDT--------
K16 3253 aa                  ---------MWDCMLVGDVSCIPFFLATVVIGNF VVLNLFLALLLSNFGSSSLSAPTADNDT--------
K17 3253 aa                  ---------MWDCMLVGDVSCIPFFLATVVIGNF VVLNLFLALLLSNFGSSSLSAPTADNDT--------
K18 3253 aa                  ---------MWDCMLVGDVSCIPFFLATVVIGNF VVLNLFLALLLSNFGSSSLSAPTADNDT--------
K19 3253 aa                  ---------MWDCMLVGDVSCIPFFLATVVIGNF VVLNLFLALLLSNFGSSSLSAPTADNDT--------
K20 3253 aa                  ---------MWDCMLVGDVSCIPFFLATVVIGNF VVLNLFLALLLSNFGSSSLSAPTADNDT--------
K21 3253 aa                  ---------MWDCMLVGDVSCIPFFLATVVIGNF VVLNLFLALLLSNFGSSSLSAPTADNDT--------
K22 3253 aa                  ---------MWDCMLVGDVSCIPFFLATVVIGNL VVLNLFLALLLSNFGSSSLSAPTADNDT--------
K23 3253 aa                  ---------MWDCMLVGDVSCIPFFLATVVIGNL VVLNLFLALLLSNFGSSSLSAPTADNDT--------
K24 3253 aa                  ---------MWDCMLVGDVSCIPFFLATVVIGNL VVLNLFLALLLSNFGSSSLSAPTADNDT--------
K25 3253 aa                  ---------MWDCMLVGDVSCIPFFLATVVIGNL VVLNLFLALLLSNFGSSSLSAPTADNDT--------
K26 3253 aa                  ---------MWDCMLVGDVSCIPFFLATVVIGNL VVLNLFLALLLSNFGSSSLSAPTADNDT--------
K27 3253 aa                  -------------MLVGDVSCIPFFLATVVIGNL VVLNLFLALLLSNFGSSSLSAPTADNDT--------
K28 3253 aa                  ---------MWDCMLVGDVSCIPFFLATVVIGNL VVLNLFLALLLSNFGSSSLSAPTADNDT--------
K29 3253 aa                  ---------MWDCMLVGDVSCIPFFLATVVIGNL VVLNLFLALLLSNFGSSSLSAPTADNDT--------
K30 3253 aa                  ---------MWDCMLVGDVSCIPFFLATVVIGNS VVLNLFLALLLSNFGSSSLSAPTADNDT--------
K31 3253 aa                  ---------MWDCMLVGDVSCIPFFLATVVIGNF VVLNLFLALLLSNFGSSSLSAPTADNDT--------
K32 3253 aa                  ---------MWDCMLVGDVSCIPFFLATVVIGNF VVLNLFLALLLSNFGSSSLSAPTADNDT--------
K33 3253 aa                  ---------MWDCMLVGDVSCIPFFLATVVIGNF VVLNLFLALLLSNFGSSSLSAPTADNDT--------
K34 3253 aa                  ---------MWDCMLVGDVSCIPFFLATVVIGNF VVLNLFLALLLSNFGSSSLSAPTADNDT--------
K35 3253 aa                  ---------MWDCMLVGDVSCIPFFLATVVIGNF VVLNLFLALLLSNFGSSSLSAPTADNDT--------
K36 3253 aa                  ---------MWDCMLVGDVSCIPFFLATVVIGNF VVLNLFLALLLSNFGSSSLSAPTADNDT--------
K37 3253 aa                  ---------MWDCMLVGDVSCIPFFLATVVIGNL VVLNLFLALLLSNFGSSSLSAPTADNDT--------
K38 3253 aa                  ---------MWDCMLVGDVSCIPFFLATVVIGNL VVLNLFLALLLSNFGSSSLSAPTADNDT--------
K39 3253 aa                  ---------MWDCMLVGDVSCIPFFLATVVIGNL VVLNLFLALLLSNFGSSSLSAPTADNDT--------
K40 3253 aa                  ---------MWDCMLVGDVSCIPFFLATVVIGNF VVLNLFLALLLSNFGSSSLSAPTADNDT--------
K41 3253 aa                  ---------MWDCMLVGDVSCIPFFLATVVIGNF VVLNLFLALLLSNFGSSSLSAPTADNDT--------
K42 3253 aa                  ---------MWDCMLVGDVSCIPFFLATVVIGNF VVLNLFLALLLSNFGSSSLSAPTADNDT--------
K43 3253 aa                  ---------MWDCMLVGDVSCIPFFLATVVIGNF VVLNLFLALLLSNFGSSSLSAPTADNDT--------
K44 3253 aa                  ---------MWDCMLVGDVSCIPFFLATVVIGNF VVLNLFLALLLSNFGSSSLSAPTADNDT--------
K45 3253 aa                  ---------MWDCMLVGDVSCIPFFLATVVIGNF VVLNLFLALLLSNFGSSSLSAPTADNDT--------
K46 3253 aa                  ---------MWDCMLVGDVSCIPFFLATVVIGNL VVLNLFLALLLSNFGSSSLSAPTADNDT--------
K47 3253 aa                  ---------MWDCMLVGDVSCIPFFLATVVIGNL VVLNLFLALLLSNFGSSSLSAPTADNDT--------
K48 3253 aa                  ---------MWDCMLVGDVSCIPFFLATVVIGNL VVLNLFLALLLSNFGSSSLSAPTADNDT--------
K49 3253 aa                  ---------MWDCMLVGDVSCIPFFLATVVIGNL VVLNLFLALLLSNFGSSSLSAPTADNDT--------
K50 3253 aa                  ---------MWDCMLVGDVSCIPFFLATVVIGNF VVLNLFLALLLSNFGSSSLSAPTADNDT--------
K51 3253 aa                  ---------MWDCMLVGDVSCIPFFLATVVIGNL VVLNLFLALLLSNFGSSSLSAPTADNDT--------
K52 3253 aa                  ---------MWDCMLVGDVSCIPFFLATVVIGNF VVLNLFLALLLSNFGSSSLSAPTADNDT--------
K53 3253 aa                  ---------MWDCMLVGDVSCIPFFLATVVIGNF VVLNLFLALLLSNFGSSSLSAPTADNDT--------
K54 3253 aa                  ---------MWDCMLVGDVSCIPFFLATVVIGNF VVLNLFLALLLSNFGSSSLSAPTADNDT--------
K55 3253 aa                  ---------MWDCMLVGDVSCIPFFLATVVIGNF VVLNLFLALLLSNFGSSSLSAPTADNDT--------
K56 3253 aa                  ---------MWDCMLVGDVSCIPFFLATVVIGNF VVLNLFLALLLSNFGSSSLSAPTADNDT--------
K57 3253 aa                  ---------MWDCMLVGDVSCIPFFLATVVIGNL VVLNLFLALLLSNFGSSSLSAPTADNDT--------
K58 3253 aa                  ---------MWDCMLVGDVSCIPFFLATVVIGNF*VVLNLFLALLLSNFGSSSLSAPTADNDT--------
K59 3253 aa                  ---------MWDCMLVGDVSCIPFFLATVVIGNL VVLNLFLALLLSNFGSSSLSAPTADNDT--------
K60 3253 aa                  ---------MWDCMLVGDVSCIPFFLATVVIGNL VVLNLFLALLLSNFGSSSLSAPTADNDT--------
K61 3253 aa                  ---------MWDCMLVGDVSCIPFFLATVVIGNL VVLNLFLALLLSNFGSSSLSAPTADNDT--------
K8 3253 aa                   ---------MWDCMLVGDVSCIPFFLATVVIGNF*VVLNLFLALLLSNFGSSSLSAPTADNDT--------
```
